# Asymptomatic carriage of *Streptococcus pneumoniae* detected by qPCR on the palm of hands of populations in rural Senegal

**DOI:** 10.1371/journal.pntd.0006945

**Published:** 2018-12-10

**Authors:** Codou Ndiaye, Hubert Bassene, Jean-Christophe Lagier, Didier Raoult, Cheikh Sokhna

**Affiliations:** 1 Aix Marseille Univ, IRD, AP-HM, Microbes Evolution Phylogeny and Infections (MEPHI), IHU-Mediterranée Infection, Marseille, France; 2 UMR VITROME, Campus International IRD-UCAD de l’IRD, Dakar, Senegal; 3 Aix-Marseille Univ, IRD, AP-HM, SSA, IHU-Méditerranée Infection, UMR Vecteurs-,InfectionsTropicales et Mediterranéennes (VITROME), Marseille, France; QIMR Berghofer Medical Research Institute, AUSTRALIA

## Abstract

Aside from malaria, infectious diseases are an important cause of death in sub-Saharan Africa and continue to pose major public health problems in African countries, notably pneumonia. *Streptococcus pneumoniae* remains the most common bacterial cause of pneumonia in all age groups. The skin is one of the main infection sites followed by the oropharynx. The skin carriage of certain pathogenic bacteria such as *S*. *pneumoniae* is often ignored or under-diagnosed. Finally, the mode of transmission of these infections remains uncertain. Here, we hypothesized that skin could play a role in the transmission of these infections. We collected 649 cotton swabs from a healthy population in Dielmo and Ndiop, rural Senegal. The sampling was carried out on the palm of the hands. After DNA extraction and actin control, qPCR targeting eight different bacteria was performed on 614 skin samples. We detected *Streptococcus pneumoniae* in 33.06% (203/614), *Staphylococcus aureus* in 18.08% (111/614) and *Streptococcus pyogenes* in 1.95% (12/614) of samples. A skin *S*. *pneumoniae* carriage was detected in more than a third of a rural population in rural Africa, highlighting the need to develop hand disinfection programs in order to reduce the burden of infections.

## Author summary

Infectious diseases are one of the leading causes of morbidity and mortality in the world. They kill nearly 17 million people worldwide each year, mainly in developing countries. They are transmitted through four main channels: air, oral, parenteral and contact. The prevention of infectious diseases requires an understanding of the population's way of life and the knowledge of pathogenic microorganisms in circulation. Many diseases are transmitted through contact with soiled hands. This study allowed us to explore the pathogenic bacteria carriage on the skin in a rural population, following skin swabs made on the palms of the hands. Previous studies have shown that hands play an important role in the transmission and the prevention of infectious diseases such as diarrhea and pneumonia. One of the main results is the detection in high proportion of pathogenic bacteria such as *Staphylococcus aureus* and especially *Streptococcus pneumoniae*. Life in rural areas exposes the population to a lot of pathogenic microorganisms. These results could be used to implement prevention strategies against certain infectious diseases, by raising awareness of the importance of body hygiene and specifically hands hygiene, in order to improve the health of the population.

## Introduction

Infectious diseases are the most important cause of death in sub-Saharan Africa [1, 2] and continue to pose major public health problems in African countries. Globally and collectively they account for 20% of the mortality in all age groups (and 33% of the mortality in the least developed countries) and 50% of infant mortality [3]. A study performed in Karachi, Pakistan found that 41% of deaths of children under 5 year were due to diarrhea and 15% to acute respiratory infections which include pneumonia [4]. The pathogenic role of *Streptococcus pneumoniae* in pneumonia, otitis media, bacteremia and meningitis is undisputed. However, its isolation on the skin is an unusual discovery with a difficult clinical interpretation [5] which can range from simple colonization in immunocompetent hosts to severe infection in patients with different underlying conditions [5–6]. In a study performed in 2014, Fenollar et *al*. reported that some bacteria that cause fever in Africa such as *Staphylococcus aureus*, *Streptococcus pyogenes* and *Streptococcus pneumoniae* are neglected in Senegal [7]. The monitoring of the carriage of these bacteria is important for several reasons. First, colonization in healthy individuals is a prerequisite for developing invasive and non-invasive diseases, and reduced colonization has been correlated with decreased pneumococcal and staphylococcal infection rates [8–9]. Second, healthy carriers serve as reservoirs for *S*. *aureus* and *S*. *pneumoniae* transmission to others in the community and in the hospital [10–11]. *S*. *pneumoniae* is one of the major pathogens infecting humans worldwide and is the most common cause of community-acquired bacterial pneumonia and otitis media, but can also give rise to severe cases of meningitis and sepsis [12]. Approximately 1.6 million people die each year from pneumococcal diseases [12]. The most frequently bacteria isolated in acute respiratory infections are *S*. *pneumoniae* and *Haemophilus influenzae*, which can occur secondarily following primary infection due to viral pathogens. Despite causing severe diseases, the asymptomatic carriage of *S*. *pneumoniae* in the nose, nasopharynx and throat was also reported. The isolation rates of *S*. *pneumoniae* obtained by nasal and nasopharyngeal (NP) sampling are similar in children, but higher than that of oropharyngeal sampling [13,14]. Its prevalence in nasopharyngeal samples varies from 7 to 99% and depends on the age, health, and socioeconomic status of the study population [15]. In Senegal, few studies on the viral and bacterial etiology of respiratory tract infections are available in pediatric settings [16]**.** Most of the publications on the prevalence of *S*. *pneumoniae* in NP samples concerned young children. Indeed, in 2003, Echave et *al*. reported a prevalence rate of 56% in Senegal [17]. However, Baylet et *al*., in 1983, reported higher NP carriage prevalence rates in rural (77%) and urban (69%) areas in Senegal and other African countries [18].The study conducted by Mediannikov et *al*.in 2014, in Senegal revealed a skin carriage of *S*. *aureus* (21.7% %), *S*. *pneumoniae* (5%) and *S*. *pyogenes* (5%) on the skin of healthy people leaving in rural areas [19].

Here, we studied the skin carriage of major pathogenic bacteria such as *S*. *pneumoniae*, *S*. *aureus* and *S*. *pyogenes* in the populations of Dielmo and Ndiop, two rural villages. In an ancillary study, we tested the skin carriage of *R*. *felis*, *B*. *crocidurae*, *T*. *whipplei*, *B*. *quintana* and *C*. *burnetii* [19–22] which have been described as causes of fever in this rural area.

## Materials and method

### Ethics statement

Before inclusion in the study, written informed consent was required from all adult participants (≥ 18 years) and from parents or legal guardians of minors (≤ 18 years). An information document that clearly explains the risks and benefits associated with the participation to the study was handed over to each patient. This sheet states the reasons and the purpose of the sampling in the presence of a parent / guardian or guarantor. The consent form stipulates that samples will not be used in the future in other studies not related with the present work without preliminary agreement of the Senegalese national Ethics Committee. Consent was obtained from each individual, and the study was approved by the national Ethics Committee of Senegal (N° 53 / MSAS / DPRS / CNERS du 31 mars 2015).

### Study site

Dielmo and Ndiop are two villages located about 280 km Southeast of Dakar, near the Gambian border in an area of Sudan-type savannah. About 700 inhabitants have been included in an epidemiological study of malaria since 1990 and monitored daily for fever and illness; the detection of cases is both active and passive. The geographical and epidemiological characteristics of the Dielmo village have been previously described in detail elsewhere [23,24], most of the houses are built in the traditional style with mud walls and thatched roofs. The main source of drinking water of the population is underground water.

### Study population

The study population consisted of people residing in the two villages, participating in the epidemiological follow-up of Dielmo and Ndiop, adhering to the principle of the project and having given their consent to participate in the study and thus provide a swab. Sampling was carried out on the palm of the hands (the right and left palm of unwashed hands) of a healthy population, all categories of age were included. It was performed in January in order to make an inventory of the skin carriage of the targeted pathogens. In addition, the sampling was done at the same location in each study participant.

### Swabs and samples analysis

Swabs are performed on the hands, after moistening the swab with sterile physiological serum. The swabs obtained are immersed in an individual tube containing 600 μl of 1X Phosphate Buffer Saline (PBS, OXOID LIMITED, HAMPSHIRE, ENGLAND). Once impregnated, the swab is pressed against the edges of the tube to release the sample, then 200 μl of the swab suspension was taken for DNA extraction using the CTAB 2% method.

### DNA extraction

To extract the DNA, 200μl of bacterial suspension from the swab was mixed with 180μl of 2% Cetyl Trimethyl Ammonium Bromide (CTAB) [25]. The mixture was incubated in a water bath at 65°C for 1 hour. Two hundred microliters of chloroform were added to the mixture, and the supernatant was recovered after centrifugation at 12,000 rpm for 5 min. The nucleic acids were precipitated by 200 μl of isopropanol after15 min of centrifugation at 12,000 rpm. The pellet was then dried in a speed vac for 3–4 min and resuspended in 200 μl RNase-free water. The DNA solution was stored in the refrigerator at 8°C until further use and the PCR was done 24 hours after DNA extraction, as DNA cannot deteriorate after only 24 hours of storage. After the PCR, the DNA was conserved at -20°C.

### DNA amplification

Except for *Bartonella*, bacteria were detected using a first intent PCR, when a specimen was tested positive in the first assay, the result was confirmed by a second quantitative PCR. A positive sample was defined as 2 positive quantitative PCR results in assays targeting 2 different repeated DNA sequences. We performed all PCR reactions in a CFX 96 thermal cycler (Biorad). Each reaction was performed at a final volume of 20 μl, containing, 10μl of polymerase TAKYON, 1μl of each primer, 1μl of probe, 2μl of RNase-free water and 5μl DNA. A positive and negative control was included in each experiment. DNA extracted from the swabbing of a healthy person in Dakar was used as negative control. The positive control consists of a suspension made from a swabbing of healthy person, in which bacterial cultures were added. The strains used as positive control are available on the « Collection de Souches de l'Unité des Rickettsies (CSUR, WDCM 875) » under the accession number: *S*. *pneumoniae* CSURP5700, *S*. *aureus* CSURP2200 and *S*. *pyogenes* CSURP6897. For each species, about ten colonies were suspended in 200μl of PBS and DNA was extracted as mentioned above.

Molecular identification by qPCR involved pathogenic bacteria such as *S*. *pneumoniae*, *S*. *aureus*, *R*. *felis*, *B*. *crocidurae*, *T*. *whipplei*, *B*. *quintana*, *S*. *pyogenes* and *C*. *burnetii* (Table 1).

**Table 1:** Target sequences, primers and probes used in a study.

Targeted Organism	Targeted gène	Name	Sequences	References
**All Rickettsia except *R*. *typhi*/ *R*. *prowasekii***	RC0338	1029-F1	GAM AAA TGA ATT ATA TAC GCC GCA AA	[26]
		1029-R1	ATT ATT KCC AAA TAT TCG TCC TGT AC	
		Rick1029_MBP	6FAM- CGG CAG GTA AGK ATG CTA CTC AAG ATA A-TAMRA	
***R*. *felis***	Biotin syntase	R_fel0527_F	ATG TTC GGG CTT CCG GTA TG	[26]
		R_fel0527_R	CCG ATT CAG CAG GTT CTT CAA	
		R_fel0527_P	6FAM- GCT GCG GCG GTA TTT TAG GAA TGG G -TAMRA	
**All *Bartonella***	ITS	Barto_ ITS3_ F	GAT GCC GGG GAA GGT TTT C	[27]
		Barto_ ITS3_ R	GCC TGG GAG GAC TTG AA CCT	
		Barto _ITS3 _P	6FAM- GCG CGC GCT TGA TAA GCG TG -TAMRA	
**All *Borrélia***	16S	Bor_16S_3_F	AGC CTT TAA AGC TTC GCT TGT AG	[28]
		Bor_16S_3_R	GCC TCC CGT AGG AGT CTG G	
		Bor_16S_3_P	6FAM-CCG GCC TGA GAG GGT GAA CGG-TAMRA	
***B*. *crocidurae***	glpQ	Borcro_glpQ_F	CCT TGG ATA CCC CAA ATC ATC	[29]
		Borcro_glpQ_R	GGC AAT GCA TCA ATT CTA AAC	
		Brocro_glpQ_MGB_P	6FAM-ATG GAC AAA TGA CAG GTC TTAC-MGB	
***C*. *burnetii***	IS1111A	Coxbur_IS1111_0706_F	CAA GAA ACG TAT CGC TGT GGC	[20]
		Coxbur_IS1111_0706_R	CAC AGA GCC ACC GTA TGA ATC	
		Coxbur_IS1111_0706_P	6FAM-CCG AGT TCG AAA CAA TGA GGG CTG-TAMRA	
	Hyp.Protein	Coxbur_IS30A_3_F	CGC TGA CCT ACA GAA ATA TGT CC	[20]
		Coxbur_IS30A_3_R	GGG GTA AGT AAA TAA TAC CTT CTG G	
		Coxbur_IS30A_3_P	6-FAM- CAT GAA GCG ATT TAT CAA TAC GTG TAT GC-TAMRA	
***S*. *aureus***	NucA	Saur__NucA__F	TTG ATA CGC CAG AAA CGG TG	[19]
		Saur__NucA__R	TGA TGC TTC TTT GCC AAA TGG	
		Saur__NucA_MGB_P	6FAM- AAC CGA ATA CGC CTG TAC -MGB	
	*Amidohydrolase*	Saur_F	CCT CGA CAG GTA ACG CAT CA	[19]
		Saur_R	AAA CTC CTA TCG GCC GCA AT	
		Saur_P	6FAM-TGC AAT GGT AGG TCC TGT GCC CA	
***S*. *pyogenes***	hypothetical	Spyo_hypp_F	ACA GGA ACT AAT ACT GAT TGG AAA GG	[19]
		Spyo_hypp_R	TGT AAA GTG AAA ATA GCA GCT CTA GCA	
		Spyo_hypp_P	6FAM- AAAATGTTGTGTTTTAGGCACTGGCGG-TAMRA	
	MipB	Spyo_mipB_F	CCA TAC GGT TAT AGT AAG GAG CCA AA	[19]
		Spyo_mipB_R	GGC TAT CAC ATC ACA GCA ACC	
		Spyo_mipB_P	6FAM- TCAGCGCCAGCTTCAATGGC- TAMRA	
***S*. *pneumoniae***	plyN	Pneumo_plyN_F	GCG ATA GCT TTC TCC AAG TGG	[19]
		Pneumo_plyN_R	TTA GCC AAC AAA TCG TTT ACC G	
		Pneumo_plyN_P	6FAM-CCC AGC AAT TCA AGT GTT CGC CGA-TAMRA	
	lyt A	Pneumo_lytA_F	CCT GTA GCC ATT TCG CCT GA	[19]
		Pneumo_lytA_R	GAC CGC TGG AGG AAG CAC A	
		Pneumo_lytA_P	6-FAM- AGA CGG CAA CTG GTA CTG GTT CGA CAA-TAMRA	
***T*.*whipplei***	WiSP family protein (WHI2)	T_whi2_F	TGA GGA TGT ATC TGT GTA TGG GAC A	[21]
		T_whi2_R	TCC TGT TAC AAG CAG TAC AAA ACA AA	
		T_whi2_P	6FAM- GAG AGA TGG GGT GCA GGA CAG GG-TAMRA	
	WiSP family protein (WHI3)	T_whi3_F	TTG TGT ATT TGG TAT TAG ATG AAA CAG	[21]
		T_whi3_R	CCC TAC AAT ATG AAA CAG CCT TTG	
		T_whi3_P	6FAM- GGG ATA GAG CAG GAG GTG TCT GTC TGG-TAMRA	

The quality of the extraction was measured by actin (the b-actin gene amplification by quantitative PCR confirmed the quality of the extracted DNA). Any sample with a Ct number (cycle threshold) that did not exceed 35 was considered positive. This number corresponds to the ability to reveal 10–20 copies of bacterial DNA [20,21].

### Statistical analysis

Data were analyzed using Open Epi, version 3.4.1 (Centers for Disease Control and Prevention, Atlanta, GA, USA). Non-parametric values were compared using a *X*2 test. Statistical significance was defined as p < 0.05.

## Results

A total of 649 samples was collected, 246 in Dielmo and 403 in Ndiop.

Among the 649 samples tested, 614 (94.61%) were actin-positive and 35(5.4%) were actin-negative.

### Prevalence of bacteria in Dielmo and Ndiop by qPCR

We detected *S*. *pneumoniae* in 33.06% (203/614) individuals, *S*. *aureus* in 18.08% (111/614) individuals and *S*. *pyogenes* in 1.95% (12/614) individuals.

We also detected *C*. *burnetii* in 13.35% (82/614) individuals, *B*. *crocidurae* in 3.42% (21/614) individuals. However, we didn’t find any case of *T*. *whipplei*, *B*. *quintana* or *R*. *felis* (Table 2).

**Table 2:** Results of prevalence of pathogenic bacteria in Dielmo and Ndiop.

Bacterium identified	Incidences (%)
*Streptococcus pneumoniae*	33.06% (203/614)
*Coxiella burnetii*	13.35% (82/614)
*Staphylococcus aureus*	18.08% (111/614)
*Borrelia crocidurae*	3.42% (21/614)
*Streptococcus pyogenes*	1.95% (12/614)

We observed that *S*. *pneumoniae* and *S*. *aureus* were the two predominantly isolated bacteria out of eight targeted bacteria, and tried to see their incidence according to age and sex.

### Prevalence of *S*. *pneumoniae* and *S*. *aureus* by age group in Dielmo and Ndiop

In Dielmo and Ndiop *S*. *pneumoniae* DNA was detected in all age groups with percentages greater than 20%. *S*. *pneumoniae* DNA was mostly detected in the youngest age groups of 0–5 and 5–10 years with respectively 39.40% (47/119) and 47.93% (58/121). The lowest rates, 17.02% (8/47) was obtained in the age groups of 45–60 years. *S*. *aureus* was detected mostly in the 5–10, 10–15 and 15–30 years age groups with 19.83% (24/121), 22.89% (19/83) and 19.55% (26/133), respectively, and in a minority in the +60-years groups, with 8.82% (3/34). Significant difference was noticed only in the 0–5 and 5–10 years age groups (Table 3).

**Table 3:** Results of prevalence of pathogenic bacteria in Dielmo and Ndiop by Age-group.

Age group (years)	*S*. *pneumoniae*	*S*. *aureus*	*P &* ᵡ^2^
**0–5**	39.40% (47/119)	15.96% (19/119)	*P* = 0.0000001 (ᵡ^2^ = 48.11, df = 1)
**5–10**	47.93% (58/121)	19.83% (24/121)	*P* = 0.000007 (ᵡ^2^ = 20.1, df = 1)
**10–15**	37.34% (31/83)	22.89% (19/83)	*P* = 0.063 (ᵡ^2^ = 3.5, df = 1)
**15–30**	23.30% (31/133)	19.55% (26/133)	*P* = 0.5 (ᵡ^2^ = 0.36, df = 1)
**30–45**	25.97% (20/77)	15.58%(12/77)	*P* = 0.16 (ᵡ^2^ = 1.93, df = 1)
**45–60**	17.02% (8/47)	17.02% (8/47)	*P* = 0.78 (ᵡ^2^ = 0.07, df = 1)
**+60**	23.52% (8/34)	8.82% (3/34)	*P* = 0.18 (ᵡ^2^ = 1.7, df = 1)

### Prevalence of *S*. *pneumoniae* and *S*. *aureus* by sex in Dielmo and Ndiop

Studies on these samples from 614 villagers in Dielmo and Ndiop showed a skin carriage in 46.74% (115 /246) of men and 23.91% (88/368) of women for *S*. *pneumoniae*, in 14.22% (35/246) of men and 20.65% (76/368) of women for *S*. *aureus*. Statistical analysis showed a significant difference between men and women for *S*. *pneumoniae* (p <0.0000001) but for *S*. *aureus* no significant difference between men and women was observed (p = 0.05485) (Table 4).

**Table 4:** Results of prevalence of pathogenic bacteria in Dielmo and Ndiop by sex.

	Men	Women	*P &* ᵡ^2^
***S*. *pneumoniae***	46.74% (115 /246)	23.91% (88/368)	*p* <0.0000001 (ᵡ^2^ = 33.71, df = 1)
***S*. *aureus***	14.22% (35/246)	20.65% (76/368)	*p* = 0.05485 (ᵡ^2^ = 3.687, df = 1)

## Discussion

In our study, we attempted to identify the prevalence of skin carriage of specific bacteria in a generally healthy rural population in Senegal. We are confident of our results because the validity of the data reported in this study is based on strict experimental procedures and positive and negative controls. The sampling was correct because 95% of samples were actin positive. Molecular analysis were carried out in two villages Dielmo and Ndiop. The most common pathogens detected were *S*. *pneumoniae* and *S*. *aureus*. They represented 51.14% of the pathogenic bacteria identified on the skin. To our knowledge, this study is the first attempt to investigate the presence of *S*. *pneumoniae* and *S*. *aureus* in the skin of asymptomatic peoples in Africa. We are also considering whether there is a link between identified pathogens and skin infections; pneumonia and respiratory infections.

It is important to note that the differences in bacterial diversity on the skin varies from one site to another at the inter and intrapersonal level, for example, the bacterial diversity of the forehead is lower than the diversity of the palm in each person, and this is also true for the forehead versus forearm communities [30]. For our study, we found it more interesting to swab the palms of the hands because we believe that it is the most likely means of bacterial transmission, through salutation, food, etc. Next to *S*. *pneumoniae* and *S*. *aureus* we detected the DNA of *B*. *crocidurae* and *C*. *burnetii*, but their known mode of transmission is not through the palms of the hands. *S*. *pneumoniae* was the most prevalent bacteria detected on the on skin. This bacterium is one of the leading causes of pneumonia in children under five years of age in Senegal [31]. *S*. *aureus* was the second most prevalent bacteria detected. The results obtained on the prevalence of this bacterium are in line with those of Mediannikov et *al*., 2014 which detected 21.7% (13/60) of *S*. *aureus* DNA on the forearm of asymptomatic populations residing in the same area [19]. We detected also *S*. *pyogenes* in the skin swabs with a smaller proportion. This rate was lower than that previously reported by Mediannikov et *al*., 2014, which showed the presence of this bacterium on the forearm of healthy populations (intact skin swabs), with a prevalence of 5% (3/60) on the same study area [19]. This difference could be explained by seasonal variation, being more common in dry than wet seasons in monsoonal climates [32]. Crowding and poor hygiene therefore increase the chance of the transmission of *S*. *pyogenes*. Also, skin infections are more frequent and are a more important cause of morbidity in overcrowded communities with poor sanitation [33]. In addition, variations in the prevalence of *S*. *pyogenes* skin infections are related to accessibility to appropriate housing and hygiene. The absence of previous work on the exploration of the skin microbiota in Africa, more specifically in sub-Saharan Africa, is a limitation for our study because these results cannot be compared to those of other countries with different climatic and environmental conditions. Most of the publications on the carriage of *S*. *pneumoniae* were done on nasopharyngeal swabs and often concerned young children [34].

The highest rates of *S*. *pneumoniae* and *S*. *aureus* were detected in the youngest age groups. *S*. *pneumoniae* was observed in the age groups 0–5 and 5–10 years. The lowest prevalence was found in age groups 45–60 years. These results could be explained by the presence of these bacteria in the environment, and those children who would be much more exposed because they take less care of their hygiene compared to adults who would be much more exposed because they take less care of their hygiene compared to adults. These results are consistent with data from a previous study that found *S*. *pneumoniae* DNA in 22% (8/36) of skin swabs from the forearm of children in the age group 0–6 years [35]. *S*. *aureus* was detected mainly in the three youngest age groups 5–10, 10–15 and 15–30 years, and in a minority in the +60-years. Just like *S*. *pneumoniae*, *S aureus* carriage was affected by age (peak prevalence at youngest age groups). A relationship could be made between *S*. *pneumoniae* infections in these villagers, a previous study on influenza like illnesses (ILI) had found that the incidence rates differed significantly between age groups, and were highest in the [6–24 month) and [0–6 month) age groups [36]. These results are comparable to ours. Children under five years of age have a higher incidence of *S*. *pneumoniae*. Finally, in these villages we have set up a field laboratory for the diagnosis of infectious diseases using the molecular biology method [22]. This technical device allows the rapid diagnosis and monitoring of infectious diseases for which laboratory analyses were generally too late to guide therapy [22]. The results show that *S*. *pneumoniae* infections are mainly localized in children under five years of age.

*S*. *pneumoniae* causes morbidity and mortality in young children, the elderly and immunodeficient patients [37], but asymptomatic carriage is more common in children. Most publications on *S*. *pneumoniae* vaccine research target the youngest age groups. Children are considered an important vector for the spread of this microorganism in the community, and preventing the carriage of pneumococci could therefore reduce the prevalence of infections. Pneumococcal conjugate vaccination protects young children against invasive diseases with *S*. *pneumoniae* [38]. Researchers have showed that the carriage rate of S. pneumoniae is low in adults compared with children because the prevalence rate, risk factors for carrying and factors promoting the spread of the organism are limited in adults [39]. The main result of our molecular analysis is the carriage of *S*. *pneumoniae* on the skin in asymptomatic people. *S*. *pneumoniae* is found all over the world. The incidence of infection is higher in children under 2 years of age and adults over 60 years of age [40]. It belongs to the family *Streptococcaceae*. It is a Gram positive bacterium. There are about 90 serotypes; the capsule surrounding the pneumococcus is the main virulence factor [41]. This bacterium generally colonizes the mucosal surfaces of the nasopharynx and upper respiratory tract, and symptoms of inflammation appear when the bacterium migrates to the sterile parts of the respiratory tract [42]. It is transmitted by infectious cells that can be spread by aerosolized microdroplets sprayed during coughing or sneezing, or by oral contact from one person to another [41]. The nasopharynx is the only documented niche for *S*. *pneumoniae* in humans, many researchers have speculated on skin colonization following reports of pneumococcal skin and soft tissue infections in adults and children in the absence of prior systemic disease [5,6,43]. However, the rather high detection rates of *S*. *pneumoniae* in skin samples in this study suggest a possible existence of a true reservoir of this pathogen on the skin. The carriage rates of *S*. *pneumoniae* reported in previous studies strongly depended on the social, demographic and medical risk factors of the study subjects, as well as the methodological variations in the methodology used. Finally, the sampling sites may vary from one study to another [38]. We have not attempted to associate our prevalence rates of carriage with specific characteristics of the population. In this study, we used qPCR testing to determine the overall prevalence of major pathogens, which had been previously isolated in other types of samples and in the same area, in febrile or healthy subjects [19]. For *S*. *pneumoniae*, the highest rate of nasopharyngeal colonization has been shown to occur at an early stage of life [11–19]. This corresponds to the low number of *S*. *pneumonia* carriers in our study, as only adult individuals were sampled.

In this study, we found that 46.74% (115 /246) of men and 23.91% (88/368) of women carried *S*. *pneumoniae* in their skin samples and statistical analysis showed a significant difference between men and women for the carriage of *S*. *pneumoniae* (p <0.0000001). Our findings are in line with a previously published study on the carriage of *S*. *pneumoniae* among older adults in Indonesia [44]. In addition, this is the first time, to our knowledge, that *S*. *pneumoniae* has been detected in the palm of hands in Senegal. We found that men and women carried *S*. *aureus*, and statistical analysis showed no significant difference between men and women for the carriage of *S*. *aureus* (*p* = 0.05485). Our results differ from those of two previous studies that showed that *S*. *aureus* carriage varies according to the sex and is higher in men [45,46]. To our knowledge, only viral respiratory infections have been studied in Dielmo and Ndiop. Available information shows that respiratory infections due to influenza (flu) viruses are more frequent [47]. From 2012 to 2013, the overall flu incidence density rate was 19.2 per 100 person-years. The flu incidence density rates were significantly different between age groups, the highest being in the [6–24 months) age group (30.3 to 50.7 per 100 person years) [36].

PCR-based techniques suffer from possible biases due to the state of the bacteria (dead bacteria) [43,48]. According to Anna Engelbrektson and *al*., 2010, molecular-based approaches do not distinguish between living bacteria and dead bacteria, so this can lead to the detection of an excessive number of pathogens by qPCR [43,48]. The culture of *S*. *pneumoniae* from skin swabs that we made in parallel with the PCR would confirm more accurately the real existence of this bacterium on the skin.

## Conclusion

In our study, we tried to demonstrate the existence of the target pathogens on the skin of people in our generally healthy study population in two villages of rural Senegal and to evaluate their impact in our two study villages using qPCR. Molecular analysis in Dielmo and Ndiop showed a high prevalence of *S*. *aureus* and *S*. *pneumoniae* carriage, especially among the youngest age groups. Our results suggest that random samples of skin swabs may contain *S*. *aureus* and *S*. *pneumoniae*. In addition, in asymptomatic subjects, we can detect the presence of certain pathogens by qPCR. In rural areas, the economic context and daily activities make this part of the population particularly vulnerable to infectious diseases. These populations live in poverty, the majority of whom are farmers and livestock breeders. As a result, they are less involved in their personal hygiene and food and household hygiene, which would lead to a considerable increase in the prevalence of infectious diseases. Fortunately, previous studies and WHO recommendations showed that body hygiene and more specifically hand hygiene could lead to a significant reduction in the prevalence of these diseases [44,49].

It appears necessary to undertake a so-called ‘‘soap project” study in Dielmo and Ndiop villages in order to evaluate the effectiveness of body hygiene in the prevention of infectious diseases.

## References

[1] World Health Statistics of the WHO (2012) WHO Technical Report Series. Geneva: WHO Available: http://who.int/gho/publications/world_health_statistics/EN_WHS2012.

[2] Adjuik M, Smith T, Clark S, Todd J, Garrib B, et al (2006) Cause-specific mortality rates in sub-Saharan Africa and Bangladesh. Bull World Health Organ 84: 181–188. 16583076 10.2471/blt.05.026492 PMC2627285

[3] World Health Organization. Engaging for Health (2006) Eleventh general programme of work 2006–2015: a global health agenda. Available:http://whqlibdoc.who.int/publications/2006/GPW_eng.

[4] Luby S-P, Agboatwalla M, Feikin D-R, Painter J, Billhimer W et al (2005) Effect of handwashing on child health: a randomised controlled trial. Lancet 2000–2005; 366 (9481): 225–33. 10.1016/S0140-6736(05)66912-7 16023513

[5] Kalima P, Riordan T(2001) Streptococcus pneumoniae: a rare ski pathogen? J Infect 42(3): 210–212. 10.1053/jinf.2001.0817 11545556

[6] Garcia-Lechuz J.M, Cuevas O, Castellares C, Perez-Fernadez C, Cercenado E et al (2007) Streptococcus pneumoniae skin and soft tissue infections: characterization of causative strains and clinical illness. Clin Microbiol Infect Dis. 26(4): 247–253. 10.1007/s10096-007-0283-7 17372776

[7] Fenollar F, Mediannikov O, Sokhna C S, Tall A., Raoult D et al (2014) Staphylococcus aureus, Streptococcus pneumoniae, and Streptocccus pyogenes DNA are common in febrile patients in Senegal. *International Journal of Infectious Diseases* 21S(1) page 337 10.1016/j.ijid.2014.03.1115

[8] Bogaert D, De Groot R, Hermans PW (2004) Streptococcus pneumoniae colonisation: the key to pneumococcal disease. *Lancet Infect Dis* 4(3):144–154. 10.1016/S1473-3099(04)00938-7 14998500

[9] Kenner J, O’Connor T, Piantanida N, Fishbain J, Eberly B et al (2003) Rates of carriage of methicillin resistant and methicillin-susceptible Staphylococcus aureus in an outpatient population. *Infect Control Hosp Epidemiol* 24(6):439–444. 10.1086/502229 12828322

[10] Wertheim HF, Melles DC, Vos MC, vanLeeuwen W, vanBelkum A, Verbrugh HA, Nouwen JL (2005) The role of nasal carriage in Staphylococcus aureus infections. Lancet Infect Dis 5(12):751–762. 10.1016/S1473-3099(05)70295-4 16310147

[11] Bogaert D, van Belkum A, Sluijter M, Verbrugh HA, Hermans PW et al (2004) Colonisation by Streptococcus pneumoniae and Staphylococcus aureus in healthy children. *Lancet* 363(9424):1871–1872. 10.1016/S0140-6736(04)16357-5 15183627

[12] Valenzuela MT, O’Loughlin R, De La Hoz F, Flannery B, De Quadros CA (2009) The burden of pneumococcal disease among Latin American and Caribbean children: review of the evidence. *Rev Panam Salud Pública* 25(3):270–279. 19454155 10.1590/s1020-49892009000300011

[13] Greenberg D, Broides A, Blancovich I, Peled N, Givon-Lavi N, Dagan R (2004) Relative importance of nasopharyngeal versus oropharyngealsamplingforisolation of Streptococcus pneumoniae and Haemophilus influenzae from healthy and sick individuals varies with age. *J Clin Microbiol* 42(10):4604–4609. 10.1128/JCM.42.10.4604-4609.2004 15472316 PMC522367

[14] Rapola S, Salo E, Kiiski P, Leinonen M, Takala AK (1997) Comparison of four different sampling methods for detecting pharyngeal carriage of Streptococcus pneumoniae and Haemophilus influenzae in children. J Clin Microbiol 35(5):1077–1079. 9114384 10.1128/jcm.35.5.1077-1079.1997 PMC232706

[15] Quintero B, Araque M, van der Gaast-de Jongh C, Vielma S, Hermans P. W. M et al (2011) Epidemiology of Streptococcus pneumoniae and Staphylococcus aureus colonization in healthy Venezuelan children? *Eur J Clin Microbiol Infect Dis* 30:7–19; 10.1007/s10096-010-1044-6 20803226 PMC2998637

[16] Marom T, Alvarez-Fernandez PE, Jennings K, McCormick DP, Chonmaitree T et al (2014) Acute bacterial sinusitis complicating viral upper respiratory tract infection in young children. *J Pediatr Infect Dis* 33:803–808. 10.1097/INF.0000000000000278 PMC4165747 24717966

[17] Echave P, Bille J, Talla I, Vaudaux B, Gehri M et al (2003) Percentage, Bacterial Etiology and Antibiotic Susceptibility of Acute Respiratory Infection and Pneumonia among Children in Rural Senegal. *J Trop Pediatr*, 49:28–32. 10.1093/tropej/49.1.28 12630717

[18] Baylet R, Diop S, Dauchy S, Roulet H, Baylet JF (1983) Infections bactériennes et virales à transmission RP dans deux populations Sérères. Bull Soc Path Ex, 76:16–20. 6839404

[19] Medainnikov O, Socolovschi C, Million M, Sokhna C, Bassene H et al (2014) Molecular identification of pathogenic bacteria in eschars from acute febrile patients, Senegal. Am J Trop Med Hyg, 91(5): 1015–1019. 10.4269/ajtmh.13-0629 25200258 PMC4228867

[20] Mediannikov O, Fenollar F, Socolovschi C, Diatta G, Bassene H, et al (2010) Coxiella burnetii in humans and ticks in rural Senegal. PLoS Negl Trop Dis 4: e654 10.1371/journal.pntd.0000654 20386603 PMC2850317

[21] Fenollar F, Mediannikov O, Socolovschi C, Bassene H, Diatta Get al. (2010) Tropheryma whipplei Bacteremia during Fever in Rural West Africa. Clin Infect Dis, 51(5):515–21. 10.1086/655677 20658941

[22] Sokhna C, Mediannikov O, Fenolla F, Drancourt, Raoult D et al (2013) Point-of-Care Laboratory of Pathogen Diagnosis in Rural Senegal.*Plos Neglec Trop Di*, 7(1): e1999 10.1371/journal.pntd.0001999 PMC3547848 23350001

[23] Rogier C, Trape JF (1995) Study of premonition development in holo-and meso-endemic malaria areas in Dielmo and Ndiop (Senegal): preliminary results, 1990–1994. Med Trop (Mars) 55:71–76. 8649271

[24] Trape JF, Rogier C, Konate L, Diagne N, Bouganali H et al (1994) The Dielmo project: a longitudinal study of natural malaria infection and the mechanisms of protective immunity in a community living in a holoendemic area of Senegal. Am J Trop Med Hvg, 10.4269/ajtmh.1994.51.123 8074247

[25] Vroh BI, Harvengt L, Chandelier A, Mergeai GDu Jardin P(1996) Improved RAPD amplification of recalcitrant plant DNA by the use activated charcoal during DNA extraction,115:205–206.

[26] Socolovschi C, Mediannikov O, Sokhna C et al Rickettsia felis–associated Uneruptive 328 Fever, Senegal. Emerg Infect Dis 2010;16(7):1140–1142. 10.3201/eid1607.100070 20587190 PMC3321914

[27] Angelakis E, Rolain JM, Raoult D, Brouqui P (2011) Bartonella quintana in head louse nits. FEMS Immunol Med Microbiol 62: 244–246. 10.1111/j.1574-695X.2011.00804.x 21477003

[28] Parola P, Diatta G, Socolovschi C, Mediannikov O, Tall A, et al (2011) Tickborne relapsing Fever borreliosis, rural senegal. Emerg Infect Dis 17: 883–885. 10.3201/eid1705.100573 21529402 PMC3321757

[29] Elbir H, Henry M, Diatta G, Raoult D, Drancourt M et al(2013) Multiplex Real-Time PCR Diagnostic of Relapsing Fevers in Africa. PLOS Neglec Trop Dis, 7(1): e2042 10.1371/journal.pntd.0002042 23390560 PMC3561136

[30] Costello E K, Lauber C L, Hamady M, Gordon J I, Knight R et al(2009) Bacterial Community Variation in Human Body Habitats Across Space and Time. *Science*, 326(5960): 1694–1697. 10.1126/science.1177486 19892944 PMC3602444

[31] Ndiaye A G, Boye C S, Hounkponou E, Gueye F B, Badiane A (2009) Antimicrobial susceptibility of select respiratory tract pathogens in Dakar, Senegal. *J Infect Dev Ctries*; 3(9):660–666. 19858566 10.3855/jidc.20

[32] McDonald M L, Towers R J, Andrews R, Fagan P, Currie B J et al (2008) The dynamic nature of group A Streptococcal epidemiology in tropical communities with the hign rates of rheumatic heart disease. *Epidemiol and Infect*, 136(4):529–539. 17540052 10.1017/S0950268807008655 PMC2870827

[33] Hay R J, Johns N E, Williams H C, Dellavalle R P, Margolis D. J et al (2014) The global burden of skin disease in 2010: an analysis of the prevalence and impact of skin conditions. *The J of Investigative Dermatology*; 134 (6):1527–1534. 10.1038/jid.2013.446 24166134

[34] Cheng Immergluck L, Kanungo Schwartz A, Mcintyre A, Schrechenberger P C, Diaz P S (2004) Prevalence of Streptococcus pneumoniae and Staphylococcus aureus nasopharyngeal colonization in healthy children in the United States. Epidemiol Infect, 132(2): 159–66. 10.1017/S0950268803001791 15061489 PMC2870090

[35] Kumar S, Wang L, Kraft A, Bose M E, Tiwari S et al (2008) Dectection of 11 commun viral and bacterial pathogens causing community-acquired pneumonia or sepsis in asymptomatic patients by using a multiplex reserse transcription-PCR assay with manual (enzyme hybridization) or automated (electronic microarray) detection. *Clin Microbiol*, 46(9): 3063–3072. 10.1128/JCM.00625-08 PMC2546717 18650351

[36] Sarr F D, Niang M, Sokhna C, Spiegel A, Richard V et al (2015) Acute Febrile Illness and Influenza Disease Burden in a Rural Cohort Dedicated to Malaria in Senegal, 2012–2013. *PLoS One* 10(12): e0143999 10.1371/journal.pone.0143999 26679177 PMC4682973

[37] Leiberman A, Dagan R, Leibovitz E, Yagupsky P, Fliss DM (1999) The bacteriology of the nasopharynx in childhood. *Int J Pediatr Otorhinolaryngol*; 49 (suppl 1): S151–53. 10577795 10.1016/s0165-5876(99)00151-2

[38] Black S, Shinefield H, Fireman B, Austrian K, Edwards K et al (2000) Efficacy, safety and immunogenicity of heptavalent pneumococcal conjugate vaccine in children. *Pediatr Infect Dis J*; 19: 187–95. 10749457 10.1097/00006454-200003000-00003

[39] Brooks KJ, Paschane DM, Fisher DG, Orr SM (1998) Pneumococcal carriage in an Alaskan drug-using population. *Int J Circumpolar Health*; 57(Suppl 1):260–4. 10093286

[40] Ryan K J and Ray C G (Ed.) (2004). Medical Microbiology (4th ed.). United States of America: The McGraw-Hill Companies, 551–552. 10.1099/jmm.0.45595-0

[41] AlonsoDeValasco E, Verheul A F, Verhoef J, Snippe H (1995) Streptococcus pneumoniae: virulence factor s, pathogenesis, and vaccines. Microbiological Reviews, 59 (4):591–603. . 8531887 10.1128/mr.59.4.591-603.1995 PMC239389

[42] Kadioglu A, Weiser J N, Paton J C, Andrew P W (2008) The role of Streptococcus pneumoniae virulence factors in host respiratory colonization and Disease. Natue Reviews Microbiology, 6(4):288–301. 10.1038/nrmicro1871 18340341

[43] Newman N, Dagan R, Reuveni H, Melamed R, Greenberg D et al(2005) Superficial Skin infection caused by Streptococcus pneumoniae in children. *Pediatr Infect Dis*; 24: 937–939. 10.1097/01.inf.0000180974.44754.41 16220102

[44] Safari D, Harimurti K, Khoeri MM, Waslia L, Mudaliana S et al (2015) Staphylococcus aureus and Streptococcus pneumoniae Prevalence among Elderly Adults in Jakarta, Indonesia. *Trop Med Public Health*, 46(3):465–71. 26521520

[45] Eriksen N H, Espersen F, Rosdahl V T, Jensen K, (1995)Carriage of Staphylococcus aureus among 104 persons during a 19 month period. *Epidemiol Infect*, 115(1): 51–60. 7641838 10.1017/s0950268800058118 PMC2271555

[46] Herwald L A, Culen J I; French P, Perl T M, Hu J et al (2004) Preoperative risk factors for nasal carriage of Staphylococcus aureus. *Infect Control Hosp Epidemiol*, 25(6):481–484. 10.1086/502426 15242196

[47] Niang M N, Dosseh A, Ndiaye K, Hay A, Diop O M et al (2012) *Sentinel surveillance for influenza in Senegal*, *1996–2009*. J Infect Dis, 206 Suppl 1: 129–35. 10.1093/infdis/jis576 23169958

[48] Engelbrektson A, Kunin V, Wrighton KC, Zvenigorodsky N, Chen F et al(2010) Experimental factors affecting PCR-based estimates of microbial species richness and evenness. *Isme J*, 4(5): 642–647. 10.1038/ismej.2009.153 20090784

[49] Nteli C, Galanis P, Koumpagioti D, Poursanidis G, Panagiopoulou E et al (2014) Assessing the effectiveness of an educational program on compliance with hand hygiene in a pediatric intensive care unit. *Adv Nurs*, 7: 1–4. 10.1155/2014/704232

