# Correction: Asymptomatic carriage of *Streptococcus pneumoniae* detected by qPCR on the palm of hands of populations in rural Senegal

**DOI:** 10.1371/journal.pntd.0012559

**Published:** 2024-10-07

**Authors:** The PLOS Neglected Tropical Diseases Editors

The *PLOS Neglected Tropical Diseases* Editors issue this notice to resolve the concerns discussed in the previously published Expression of Concern on this article [1, 2].

Specifically, concerns were raised that the ethics approval number reported in [1] (N° 53/MSAS/DPRS/CNERS) was also reported in multiple other publications [3–5], and the PLOS article [1] did not include sufficient information to determine when the data collection for the study had taken place.

The Corresponding Author responded, stating that the ethics approval document reference number was correctly reported in [1–5] because these articles all report species of skin microbiota that were isolated from samples collected during the same study. In addition, the Corresponding Author clarified that the interviews and sampling reported in [1] were conducted quarterly from January to December 2016.

A representative from the Aix-Marseille Université stated that the institutional investigation into the ethics concerns concluded this article meets ethical standards. They responded to the concerns, stating that sample collection for this study was conducted in Senegal, and that the study was approved by the Comité National d’Ethique pour la Recherche en Santé (CNERS) in Senegal. Furthermore, the institute stated that as the Institut Hospitalo-Universitaire Méditerranée Infection was only involved in the analysis of the anonymous samples obtained in Senegal, French institutional ethics approval was not required for this study. The representative provided ethics approval documents N° 53/MSAS/DPRS/CNERS and N° 0271 MSAS/DPRS/DR, both issued by the Ministère de la santé et de l’action sociale (Ministry of Health and Social Action) of Senegal, for editorial review.

Both documents are issued on the protocol “*SEN 14/30*: *Evaluation de la promotion de l’hygiène corporelle dans la prévention des maladies infectieuses en milieu rural au Sénégal*: *le project Savon”*. The document N° 53/MSAS/DPRS/CNERS was issued on 31 March 2015 and provides ethics approval to carry out the same study but does not state a specific authorization period. The corresponding author stated that the approval covered the entire period of the study. The second document, N° 0271MSAS/DPRS/DR, was issued on 01 April 2015 and provides administrative authorization for the study for one year (April 2015 and April 2016).

In light of the documentation and responses provided, the final sentence of the Ethics statement in the Materials and method section of [1] is updated to:

Consent was obtained from each individual, and the study was approved by the Comité National d’Ethique pour la Recherche en Santé (CNERS) in Senegal (N° 53 / MSAS / DPRS / CNERS issued 31 March 2015 and N° 0271MSAS/DPRS/DR issued 01 April 2015). The samples and data reported in this study were collected from January to December 2016.

With this update, the *PLOS Neglected Tropical Diseases* Editors consider the ethics approval concerns resolved. This Correction supersedes the previous Expression of Concern [2].
